# To Eat or Not to Eat—A Qualitative Exploration and Typology of Restrictive Dietary Practices among Middle-Aged and Older Adults

**DOI:** 10.3390/nu15112466

**Published:** 2023-05-25

**Authors:** Lena Bandelin-Franke, Liane Schenk, Nadja-Raphaela Baer

**Affiliations:** Institute of Medical Sociology and Rehabilitation Science, Charité—Universitätsmedizin Berlin, Corporate Member of Freie Universität Berlin and Humboldt-Universität zu Berlin, Charitéplatz 1, 10117 Berlin, Germany; lena.bandelin@charite.de (L.B.-F.); liane.schenk@charite.de (L.S.)

**Keywords:** restrictive dietary practice, dietary refrain, restriction, dietary behavior, food choice, fasting, healthy ageing, qualitative research, qualitative typology

## Abstract

Favorable diets often include restrictive practices that have proven health benefits, even if initiated later in life. The aim of this qualitative study is to gain a comprehensive understanding of Restrictive Dietary Practices (RDPs) among a sample of middle-aged and older German adults (aged 59–78 years). We conducted 24 narrative in-depth interviews and analyzed the data using qualitative content analysis (Kuckartz). Following an inductive thematic approach, a typology was reconstructed comprising four typical RDP characteristics: I. The Holistically Restraining Type, II. The Dissonant-savoring Restraining Type, III. The Reactively Restraining Type, and IV. The Unintentionally Restraining Type. These types differed regarding the practical implementation of, e.g., restrictive food choice into everyday routines, barriers to do so, as well as with respect to attitudes and motives underlying RDPs. The major motives for adopting a RDP involved health, well-being, ethical, and ecological concerns. The most prominent barriers to a ‘successful’ adoption of RDPs were the enjoyment of food and the desire for spontaneity and freedom of (food) choice. Our study offers an in-depth understanding of the aspects that shape the widespread practice of dietary restriction among middle-aged and older adults. Lifeworld-related changes in RDPs and possible ‘type shiftings’ are discussed as well as the meaning and chances of RDPs for public health promotion.

## 1. Introduction

Definitions of what constitutes a healthy diet vary not only with respect to the (physiological) needs of specific populations—such as older adults [1]—but also considerably across socio-cultural contexts [2] and, over time, as scientific knowledge advances [3,4,5]. Overall, most definitions and recommendations for a healthy diet include some kind of restriction in the consumption of certain foods or nutrients. For example, the guidelines published by the World Health Organization [6] recommend a reduced or moderate intake of sugar, processed fats, and salt. While there certainly is scientific consensus on the health benefits of restricting these specific foods or nutrients, this does not readily apply to Restrictive Dietary Practices (RDPs) in general. Rather, health effects associated with RDPs appear to be two-fold. On the one hand, dietary restraint shows health-promoting benefits, such as a successful self-regulation or weight management, i.e., a congruence between the intention to and practical implementation of RDPs [7,8]. On the other hand, various negative health outcomes are evident [8,9], which are mostly associated with a tendency towards “counterregulatory eating” [7]. As a consequence, (strictly) restrained eating habits may result in an unbalanced diet, eventually leading to nutrient deficiencies and associated physiological diseases. At the same time, psychological pathologies may develop in response to (strict) RDPs, such as bulimia nervosa or obesity [10,11]—both of which also appear to result from “unhealthy self-regulation” [8].

To gain an understanding of the concept and practice of dietary restraint, a brief framing of the conceptualizations and empirical study of RDP is provided in what follows. Starting in the 1970s, dietary restraint was initially studied as a cause of eating pathologies and weight disorders from a psychological perspective [7,12]. Ever since, phenomena related to restrained eating have been subject to historical and ongoing controversy. Thereby, the scientific debate has been largely shaped by two influential explanatory models: the Restrained Eating Model [13] and the Three Factor Model of Dieting [14]. Both models differ considerably in their conceptualizations and measurements of dietary restriction [9]. The Restrained Eating Model, on whose basis the well-known Restraint Eating Scale [15] was developed, primarily addresses dietary resistance to overeating and loss of weight control. More differentially, the Three-Factor Model distinguishes three types of restrained eating. This model was developed based on the critique of the Restrained Eating Model as being unidimensional in conceptualizing restraint as a merely internally caused, “trait-based process (…)”, mainly pathologizing restrictive eating and dismissing external influences affecting RDPs [9].

Mainly originating from these models and developing them further, manifold conceptualizations and definitions of restrained eating have been proposed. Overall, dietary restraint may be defined as the “cognitive effort exerted by an individual to eat less than they would like” [14] or the intent to control food intake in line with personal eating norms [8], regardless of the behavioral outcome [7]. While dietary restraint is not strictly distinct from other diet-related practices, such as caloric restriction or dieting [12], definitional differentiations have been discussed. Therefore, dieting has been defined as a rather transitory behavioral action involving the adherence to an eating plan (mostly aiming at energy restriction). In contrast, restraint appears to be a more stable and specifically cognitive process that is not necessarily related to a restricted energy intake [8,14].

Ways of exerting dietary restraint have been distinguished between “rigid”, on the one hand, and “flexible”, on the other, referring to an adherent, complete exclusion, or a rather mutative limitation of the consumption of certain foods (usually regarding quantity) [16]. Moreover, a distinction has been made between “successful” and “unsuccessful dietary restraint”, emphasizing the potential discrepancy between behavioral intention and its adherent practical implementation [16,17]. RDPs may occur more consciously, e.g., in the form of an informed, knowledge-based consumer behavior or food choices based on moral convictions. At the same time, preconscious processes operating at a pre-reflexive level of habitual practice also play a crucial role in shaping RDPs [18]—such as in the context of so-called "hedonic appreciation" [19]. Regarding consciously exerted RDPs, motives underlying dietary restraint have been studied, with weight control being a central intention [7,8]. In the context of specific diets (e.g., low-carb or high-fat diet), weight loss as well as health promotion showed to be predominant motives to renounce specific foods [20,21]. Increasingly, environmental sustainability and animal welfare were also shown to motivate people to engage in RDPs—especially in the context of plant-based diets [15,22].

Building on the conceptual debate and the existing scientific evidence on dietary restraint and dieting, this study examines the phenomenon of RDP from a sociological–praxeological perspective [23], thereby paying attention to pre-reflexive habitual as well as consciously decided practices of dietary restraint. We define RDP as a complex set of cognitive intentions and motives, as well as the practical execution or habitual implementation of a reduction in or elimination of specific foods, meals, or portion sizes and/or a restriction of particular macronutrients (e.g., calories and fat). It may be carried out once, periodically, or persistently integrated into daily routines within particular or across several biographical phases.

To date, studies have barely addressed (un-)consciously retrieved motives to practice dietary restraint or its practical (un-)successful implementation irrespective of particular diets and concerning different types of dietary restraint among the general population. Mostly, in Psychology and Nutrition Sciences, quantitative (interventional) studies have focused on dietary restraint as part of specific dietary patterns (e.g., the Mediterranean Diet) [24,25], or among populations with pre-existing illnesses or diet-related symptoms [18,26] as well as in relation to particular personality traits [19,27]. Qualitative explorations are still scarce and have either rather broadly addressed motives for an overall change in dietary habits [28], or analyzed the benefits of and barriers to RDPs rather narrowly by focusing on particular dietary components, such as meat [29].

To the researchers’ knowledge, no (qualitative) study has yet examined RDPs independent from particular diets or diseases and within specific groups of the general populations. Specific population groups—such as people transitioning into older age (middle-aged and older adults)—have specific lifeworlds, resources, and thus particular needs, which, among others, manifest in specific dietary practices involving restraint. Hence, it is critical to study such specific population groups separately and in greater depth to gain a detailed picture of their specific practices and to draw inferences for targeted public health dietary strategies. Since basic research on dietary practices in general appears scarce among middle-aged and older adults, addressing RDPs specifically (among others) in this population is of particular epistemic relevancy.

Going along with the conceptually diverse definitions of RDPs, there is an ongoing controversy about the measurability and appropriate measurement of RDPs [7,9,12,30,31] Several measures to accurately assess various dimensions of restrained eating have been established (e.g., the Restrained Scale [13] and the DEBQ [32]) and the need to enhance the (internal) validity has been discussed [7,9,15]. However, such research activities solely concern the identification and quantitative examination of the rather broad group of “restrained eaters” [15] and lack conceptual and thus methodological differentiation. Polivy et al. [15] stressed the need to identify and distinguish between various dimensions of dietary restraint, including behavioral factors, attitudes, motives, identity-, and affect-related aspects. Understanding such aspects is specifically important with respect to health promotion strategies, since different behavioral patterns and underlying influencing factors also differently affect various health outcomes [15,33]. Surprisingly, this methodological debate has to date only focused on the quantitative assessment of RDPs, neglecting the analytical benefit and necessity to gain a deeper understanding by means of reconstructive, qualitative measures.

To fill the outlined methodical and empirical research gaps, a comprehensive in-depth understanding of RDPs and related motives and attitudes within general, non-clinical population groups is needed. Exploring various facets related to the implementation of RDPs, this study provides a qualitative typology of Restrictive Dietary Practices among a sample of middle-aged and older German adults.

## 2. Materials and Methods

As part of the German Competence Cluster NutriAct—Nutritional Intervention: Food Patterns, Behavior, and Products, a qualitative, cross-sectional study was carried out from 2016 to 2020. The study was conducted in accordance with the Declaration of Helsinki. Ethical approval was granted by the Ethical committee of the Charité—Universitätsmedizin Berlin (EA4/151/16).

### 2.1. Recruitment Procedure

Community-dwelling adults aged 50 years and older living in Germany were eligible for study participation. Recruitment was carried out by a combination of theoretical sampling [34] and snowball sampling. A heterogeneous sample was sought in terms of sociodemographic factors, such as age (within the pre-defined age group) and the current relationship status, various socialization backgrounds, as well as regarding differing experiences with the retirement status passage (e.g., retirement path). To obtain a relatively broad range of participants, both online and offline recruitment channels were used, e.g., digital social networks as well as flyers in public places, such as supermarkets and pharmacies. By that means, efforts were made to select the sites in such a way as to ensure the recruitment of participants from various social milieus.

Aiming for theoretical saturation, participant recruitment took place gradually during the analysis process, i.e., participant enrollment continued until no new insights could be expected from further interviews. Study participation was compensated with an expense allowance of EUR 20 per interview.

### 2.2. Data Collection

*n* = 24, semi-narrative in-depth interviews were conducted using a flexible interview guideline. Depending on the participant’s preference, the interviews took place either at the researchers’ institution, at the participant’s home, or in a public space. Prior to the interviews, participants provided written informed consent and verbally agreed to the interviews being tape-recorded. The tape-recorded data material was saved in encrypted form and transcribed verbatim. Thereafter, transcripts were anonymized, i.e., any information potentially revealing a study participant’s identity was pseudonymized. The data material was handled according to the European General Data Protection Regulation (GDPR). To obtain a picture of the participants’ biographical backgrounds, the first guiding question aimed at stimulating narratives and triggering thick descriptions of childhood memories, including recollections of daily mealtime routines. The major interview themes and sample guiding questions are summarized in Table 1.

### 2.3. Data Analysis

The data were analyzed thematically, following the seven steps of qualitative content analysis described by Kuckartz [35]. This approach basically includes intensive (re-)reading of the data, organizing them by writing case summaries, building a “coding frame” (ibid.), coding the data, and finally analyzing the coded data, i.e., among others, by contrasting (sub-)codes across interviews with respect to the research questions. As a result, a categorical system(see Table A1 in the Appendix A) was inductively derived from the material, by having revised and adapted the coding process multiple times. For each main theme, a number of subthemes was defined that describe the content in more depth and structure. The coding process was conducted using the MAXQDA 20 software (VERBI GmbH, Berlin, Germany).

Based on the final category system, the content-analytical development of a typology was carried out. Distinct types could be defined following Kuckartz’ approach [35] of the type building process that includes five consecutive steps: first, determination of the “attribute space” [35]; second, grouping of the individual cases and building of types; third, in-depth description of the types; and finally, the assignment of cases to the different types and analysis of their inter-relationships. The categorical system and typology building processes were continuously reviewed and discussed intersubjectively between co-authors as well as within an institutionalized research workshop of the Institute of Medical Sociology and Rehabilitation Science of the Charité—Universitätsmedizin Berlin.

## 3. Results

The majority of the participants was female (*n* = 15) and *n* = 9 interviewees were male. The mean age of the sample was 65 years (age range = 59–78 years). Participants varied with regard to their (former) professions. While some interviewees were still (partly) employed at the time of the interview (*n* = 6), others were retired for a varying length of time (*n* = 19) (for more details see Table 2). The mean length of the interviews was 69 min (range = 46–88 min).

### 3.1. General Results

Overall, various Restrictive dietary practices (RDPs) inductively surfaced from the data material (see a short version of the categorial system in Appendix A). Upon being asked about general lifestyle changes and dietary habits during the retirement status passage in particular, all participants showed dietary restraint—either as formerly executed, currently established, planned, or potential future practice. On the one hand, this was explicitly expressed and verbalized in absolute terms (e.g., “Well, I always say, I don’t eat dead animals.” (16W)) and, on the other hand, rather vaguely implied as in the following: “As an older woman, you don’t eat so much meat anymore.”(13 W). While different ways of dietary restraint were a central theme, dietary practices aiming at increasing the consumption of health-promoting foods, such as vegetables and fruit, played a rather subordinate role.

Based on such themes, four overarching dimensions were identified as constituting Dietary Restrictive Practice: (1) practical implementation of RDP, (2) attitudes towards RDP, (3) motives for RDP, and (4) barriers towards RDP. The practical implementation (1) of RDP varied from a deliberate, intentional to a habitualized, unconscious practice. Differing manifestations regarding the extent and timeframes of restriction as well as the specific objects (food products) restrained from emerged. While the various forms of restraint mostly involved the omission of presumed unhealthy foods, dietary practices that aimed to increase the consumption of health-promoting foods played a subordinate role. Main objects of dietary restriction were either specific foods (e.g., meat, sugar, and alcohol), processed foodstuffs, or those being rich in (refined) carbohydrates or fats. In particular, meat was mentioned across all interviews in this context, whereby its restrictive consumption showed to be on a spectrum from “not daily” to a vegetarian lifestyle. Regarding the timeframe, fasting or fasting-like dietary practices appeared as the strictest forms of restraint and ranged from episodic and complete food abstinence to (intermittent) fasting.

Furthermore, attitudes (2) towards RDPs were diverse, especially regarding its effect on health and wellbeing, ranging from a positive perspective based on personal experience or the external attribution of advantages as a means of self-efficacy to questioning of its benefits. Some participants expressed that there are clear benefits to RDPs with the limitation that it could, practiced in a rigid form, interfere with quality of life. Closely related to such attitudes were various motives for and barriers to engage in dietary restraint: motives for RDPs (3) concerned individual as well as societal benefits. At the individual level, the maintenance and/or improvement of health and subjective wellbeing appeared to be a major motive. Among the fasting participants, for example, (intermittent) fasting was practiced on an intentional, conscious level: “Well, I always actually feel quite good when I fast. Well, actually really good.” (5W). Closely related, weight control or weight loss were goals intended to be realized with RDPs and were sought not only for positive health effects but also for aesthetic reasons. In contrast, other fasting participants had established routines of skipping single meals without any particular motives and in a rather unconscious manner, e.g., due to taste preferences. With respect to societal benefits, the conditions of food production—particularly in terms of ecology and animal welfare—were central themes that emerged across interviews (e.g., “I mean, the consumption of large amounts of meat damages the environment and the, the air (…). A lot of resources are wasted just to produce the meat in the first place (…).“ (M10)).

In addition, barriers to initiate and/or maintain RDPs (4) surfaced with respect to an unwillingness or reluctance to engage in dietary restraint. In particular, the hedonistic enjoyment of food and following taste preferences were central counter-motives. In addition, the desire to retain everyday spontaneity and flexibility, as well as to gain freedom of choice as opposed to following set rules manifested in a reluctance to RDPs. Noteworthy, barriers were not necessarily perceived as obstacles to be overcome but were sometimes rather put forward as a deliberate decision for a limited or absent RDP. In the following, this is implicitly taken into account when referring to barriers towards RDPs.

### 3.2. Typology of Older Adults’ Restrictive Dietary Practice

Based on the above-mentioned inductively generated dimensions of RDPs, a typology could be reconstructed that reveals four different manifestations or types of engagement with RDPs: The Holistically Restraining Type (Type I), The Dissonant-savoring Restraining Type (Type II), The Reactively Restraining Type (Type III), and The Unintentionally Restraining Type (Type IV). Each of these types showed different specifications of Restrictive dietary practices. Subsequently, each type and their characterizations are described in detail with respect to the above-mentioned four dimensions and contextual factors. An overview is presented in Table 3.

#### 3.2.1. Type I: The Holistically Restraining Type

A central characteristic of The Holistically Restraining Type’s RDP is that these are based on internalized assumptions and convictions of an overarching, holistic health-oriented lifestyle and associated conceptualizations of a healthy diet. Those convictions render the restriction of foods considered unhealthy a necessity and require its integration into daily routines without compulsive adherence. As an example, (rigidly) refraining from ready-made or processed foods appears central to this type and necessitates relative high levels of engagement with foodwork:

*“I don’t want to have any pre-made stuff. I never buy any ready-made food. I buy natural produce and, where possible, I pay attention to where they come from.”* (16W)

Moreover, for Type I, restrictive practices show to be habitually embedded into long-term dietary routines and can be described as ascetic due to their fairly rigorous implementation. This becomes either apparent by participants referring to their practices with a vigorous choice of words, such as “I never buy” and “I don’t want to have any pre-made stuff”, or by them explicitly pointing at the duration of established restrictive habits (e.g., “I changed my diet in 1988 - to a vegetarian diet.” (16W)). Refraining from certain foods (e.g., meat) or food components (e.g., sugar) and, following a restrictive food choice pattern (i.e., a set of restrictions from various foods/food components) is one of three partially intertwined ways, in which dietary restraint among people of this type is practiced. Another such was regulation of the amount of energy intake, for instance, by means of “low-calorie days”:

*“And back then, I lost a massive amount of weight within three weeks and well, to maintain the weight, well, there was this recommendation to do a “hunger day” once a week, and that is what I have been doing already since 2005.”* (3W)

Here, one central motive becomes apparent, namely, weight control. The third way of RDP among Type I participants concerns the timeframes in which dietary restraint is practiced. Those timeframes varied across individuals, and the implementation of regular intervals of fasting routines showed to be one popular variant. Such a fasting routine ranged from fasting for several days per month or year to daily intermittent fasting alternating from 16 h of food abstinence to an 8 h window of potential food consumption. The following quote illustrates a typical daily fasting routine of the latter type:

*“Long story short, I do so-called intermittent fasting, which means, I always fast for 16 h after the last meal in the evening, which is unfortunately in my case usually fairly late. So, if I stopped eating at 8 o’clock in the evening, which I rarely manage to do, eight plus sixteen, would be 12 o’clock, so I could eat again at noon. […] And, when I sometimes eat at midnight, I have to get through this and then I do not eat anything until 4. It is surprisingly easy for me.”* (M19)

For this participant, who follows a clear self-imposed set of rules, dietary restriction may occasionally be challenging, but at the same time is experienced as “surprisingly” easy. The astonishment expressed in this verbalization may be rooted in a perceived divergence between a theoretically expected burden of adhering to these rules and the actual experience of their practical implementation. While his choice of words (e.g., “I have to get through it”) indicates an effort he may indeed be confronted with, this seemingly does not interfere with his strict commitment *(“always”)* to the RDP. In contrast, another participant of this type practices fasting less regularly *(“from time to time”* (W5)), for several days in a row *(“I get along very well with seven days without food”* (W5)), thereby stressing the ease of doing so.

Among participants of Type I, the central motives for dietary restraint—health, wellbeing, and weight control—appeared to be intrinsic as well as externally driven. On the one hand, personal experiences of or the desire for wellbeing and bodily comfort (e.g., weight control, see quote in lines 317–320) as well as a profound knowledge on the health effects of specific restrictive diets motivates participants to practice dietary restraint. On the other hand, the initiation of such practices is also inspired by the personal social environment:

*“Maybe 15 years ago, a colleague of mine, who really wanted to fast, told me about it. […] She gave me a book about fasting, which I read and thought, yes, ‘you want to do it, too, yes’.”* (5W)

Especially regarding the maintenance and/or improvement of health and wellbeing (e.g., avoidance of malnutrition due to veganism, and weight loss), dietary guides are studied in-depth and encourage the adoption of specific RDPs. Hence, for the Holistically Restraining Type, the motives to practice restraint are based on a conceptualization and understanding of food choices in accordance with (popular) scientific evidence. In some cases, specific nutritional concepts and corresponding dietary styles (e.g., wholefood diet) promulgated by experts were action-guiding:

*“And then I got some literature and got this book written by a mister Doctor Bruker, who also is a pioneer in vegetarian diet and, above all, in wholefood diet. And, eh, I read this book and so I knew about it in theory and I have put it into practice because it all seemed so logic to me, right?”* (16W)

Considering it “logic”, this participant is convinced of this dietary concept according to which she aligns her RDP. Similarly, another participant substantiates his episodic restrictive dietary practice against the background of a(n) evolutionary-bio-physiological logic: *“Prehistoric human beings did not constantly [eat] all day long [or] had breakfast every morning.“* (19M). Additionally, more overarching intentions were expressed with respect to a critical stance on ethical aspects: animal welfare, ecological responsibility in the context of food production (*”[M]eat consumption causes damage to the environment and the air.”* (M10)), as well as problems with the global (over-)use of natural resources (“[…] waste of very many resources” (10M)) showed to be pivotal initial motives for RDPs. Such reflections were either elicited by personal experiences *(“[T]his experience at the slaughter […] made me think.“* (16W)) or subject to a basic orientation and an ongoing engagement with politics and the news. For Type I, the major barrier to the self-applied RDP depicts the enjoyment of eating that appears to be related to a relatively pronounced taste orientation. Such orientations are often perceived as being in conflict with the intended health-oriented or weight-regulating diet. Therefore, participants anticipate feelings of failure and/or guilt if foods considered unhealthy were consumed, for instance. At the same time, Type I typically strives to prevent himself/herself from succumbing and potentially “failing” by reflecting on and controlling “tempting” circumstances. As an example, individuals would avoid preparing specific foods just for themselves as they anticipate giving into the temptation and consuming abundantly in the absence of any social control. However, occasional exceptions are not considered as failure but rather justified as a necessary etiquette within social contexts. This is exemplary illustrated in the following quote of a participant who usually refrains from breakfast:

*“Recently, I was in Mallorca with two friends of mine and there, I joined the breakfast, because it would have been uncommunicative not to do so. […] That really doesn’t set me back or so, that’s ok.”* (19M)

#### 3.2.2. Type II: The Dissonant-Savoring Restraining Type

To The Dissonant-Savoring Restraining Type, a RDP is a necessary component of a healthy lifestyle and regularly practiced—not due to an inner need, but rather out of reason. The central motives for Type II were weight control and the maintenance of physical health as well as ethical (animal welfare) and ecological aspects. With regards to the latter, participants attribute physical health benefits to RDPs particularly in the context of primary prevention, e.g., as implied below:

*“I have always eaten well, and a lot of meat, of course, which I now slowly reduce, too. […] just for health reasons because I tell myself, you don’t have to eat all that meat all the time.“* (20M)

Compared to Type I, individuals of Type II show a relative superficial diet-related knowledge and a moderate level of involvement in foodwork. Meal preparation, for instance, is usually based on spontaneous moods or special occasions rather than on fixed, internalized routines. Moreover, participants of this type engage in restrictive diets based on a general understanding of a healthy diet. Unlike Type I, this understanding is independent of any specific philosophy or (scientific) trend but rather loosely anchored in a general health orientation. In accordance with this orientation, Type II participants conceptualize dietary restraint as an ongoing consideration and trial to balance between the quantitative amount and qualitative value of food:

*“[In] my opinion, it is maybe not, so maybe […] the small amount is wrong, that is even wrong, you have to eat, but you have to eat the right things, whereby I would not claim to eat the right things, I just ty to.”* (23W)

While the definition of a “right” or “wrong” practice of dietary restraint remains imprecise here, a certain hierarchization of the quality over the quantity of food becomes apparent. Moreover, instead of adhering to a clear set of rules and aiming for specific goals, the participants of this type mostly follow a rather indistinctively defined tendency of dietary restraint (e.g., in terms of pace *(“slowly”)* and try to achieve their similarly vaguely defined objectives *(“little less*”)):

*“No, not purposefully or something, no. […] I could do with a little less [of weight] and therefore I just want to see now, if I manage […] but without a plan and without any purpose, just slowly adjust [(habits)] if possible […] I hate such things because I don’t manage to stick to it anyways. I can make a plan now, but I know exactly that if I-, if there is a delicious sausage crossing my way or a nice fish sandwich or something like that, I can’t say ‘no’, I just cannot say, ‘my plan doesn’t allow to eat that’.”* (20M)

Exemplary for Type II, this quote illustrates the desire for a flexible dietary routine involving a certain freedom of choice and a rather loose commitment to self-assigned RDPs. This participant explains his strong rejection towards the fixed rules of dietary restraint by his allegedly incapacity *(“I can’t”)* to resist food-related temptations. Here, a lack of gustatory self-regulation and/or the unwillingness to restrain becomes apparent. While this is seemingly considered a failure, others accept their non-restrictive practice as a given, natural fact to which they ascribe major importance (*“Well, if I am hungry, I have to eat.”* (13W)). The positively connoted choice of language *(“delicious sausage crossing”* and *“nice fish sandwich”* (20M)) underlines this participant’s orientation towards enjoyment and taste. This orientation shows up even stronger in cases where dietary rules are considered a threat to the quality of life:

*“I don’t take it too doggedly. […] if I am supposed to restrict myself to such an extent for a lifetime, I’d rather die early. So, this is no quality of life to me.”* (8W)

In this case, the enjoyment of food competes with restriction and is seen as an integral part of psychological well-being. As a result, savoring is likely to challenge or abandon an initial intent to restrain in a situation where desired foods are available. Hence, for Type II, the pleasure of eating and desire for flexibility and spontaneity are the predominant barriers to RDP. At the same time, the readiness and adherence to practice dietary restraint is increased if the foods ‘permitted’ to eat are regarded as tasty. In some cases, the correspondence between such ‘permitted’ foods and taste preferences even depicts a pre-requisite for taking the emotional constraints associated with a limited freedom of food choice:

*“I can torment myself if a plan tells me ‘you have to skip bread rolls, skip potatoes, skip pasta’, I can do that, but the little I [may] eat, that has to be tasty.”* (23W)

#### 3.2.3. Type III: The Reactively Restraining Type

Participants belonging to The Reactively Restraining Type show an explicit readiness to refrain from or reduce specific foods and drinks in the face of manifest illness or risk factors. As opposed to the two types mentioned, one major motive for RDP is secondary health prevention, since RDP is regarded as an effective measure to prevent the emergence of (further) disease burdens:

*“I still keep away from coffee in the morning a little bit because of my hypertension.”* (18W)

*“But when my liver enzymes were relatively bad, I even had to refrain a whole year [from] drinking [(alcohol)], back then, 3 years ago, I did not drink a sip. And afterwards less.”* (11M)

As seen above, RDP among Type III-individuals can take the form of an absolute restriction *(“not a single drop”*). At the same time, the extent of restriction may change over time: as in this example, absolute RDP only applied to the initial episode, followed by a conversion to a moderate alcohol consumption. Mostly, the practice is implemented as a conscious, moderate reduction in specific dietary components *(“a little bit”* and *“not every day a cutlet or goulash […]”*).

In contrast to Type I and partially Type II, rather than an overall health-promoting concept, merely the abstinence from single products or the reduction in calories is executed. RDP is motivated by a vague and undifferentiated understanding of (un)healthy foods, without any reference to precise health effects (e.g., *“drinking coffee in general is not healthy.”* (15M)). Participants’ food knowledge is typically selective and mostly based on anecdotes and news headlines or sometimes on recommendations from physicians. Specific sources of information are not provided, which may hint at a rather superficial dealing with the topic of interest. Contrary to Types I and II, the engagement in foodwork is limited to occasional and quick cooking and a tendency towards the consumption of fast food. Here, a central orientation underlying these dietary practices surfaced: dietary practices, such as foodwork and food consumption, are matters of convenience rather than subject to a pronounced taste orientation. At the same time, and similar to the types mentioned, the enjoyment of food appeared as one main barrier to RDP:

*“Well, for breakfast, then I unfortunately am, then I still like to eat cheese and sausage, […] because that’s not very good either, but (2) particularly cheese is not so healthy, but, well, I like to eat cheese.”* (15M)

As illustrated here, Type III participants face the difficulty of dealing with the tension between RDP and individual taste preferences. This tension seems to be perceived as exhausting, but also as guilt-inducing. In this example, this may be indicated by his regret (e.g., *“unfortunately*”) about his taste. In contrast, Type II participants would rather self-confidentially stand by their taste preferences and their decision to balance those with their RDP in order to achieve their set goals. Moreover, overlapping with the Holistically and Dissonant-Savoring Restraining Types, individuals of Type III also show a mental reluctance or even rejection towards set rules of restrictions that counteract their flexibility and freedom of choice—even if these rules were self-chosen. To avoid unpleasant constraints by strict obligations, some Type III participants would rather accept a health risk or abandon self-imposed goals as shown in the following:

*“[…] not with such an obligation, not with this: ‘I have to’. Then, I don’t want to. Then, I don’t want to. Then, I just want to live as I do.”* (14W)

#### 3.2.4. Type IV: The Unintentionally Restraining Type

Opposed to all other types, The Unintentionally Restraining Type does not refer to any conscious, intended RDP. However, this type shows a highly routinized daily eating routine (e.g., “*[T]hat [(dietary routine)] has been like that way for my entire life*.” (6M)) that certainly involves an unintentional RDP. This especially manifests itself in a habitual adherence to a pre-reflexive established plan of food choice, in which specific foods are strictly excluded and thus avoided. For example, one individual describes having the same meal every day:

*“NB: And then, [you] always [eat] two slices of brown bread and cheese again and sausage as in the morning?//Exactly. In the morning I usually eat soft cheese, Brie or (.), and in the evening one slice of cheese.“* (6M)

Major motives for this habitual dietary practice unintentionally including RDP appeared to be individual taste preferences and practicability. Moreover, as illustrated in the following, certain self-ascribed character traits and an associated need of routinized, repetitive practices emerged as a reason for these habits:

*“Because, because […] I am such a boring guy, who always needs to have the same […], who always needs to have the same, yes.“* (6M)

Contrary to Types I–III, Type IV shows little dealing with and knowledge of food-related topics in general and does not refer to any active acquisition of respective information. Furthermore, individuals belonging to this type seem to be marginally involved in foodwork. Food choice and shopping as well as meal preparation typically lie in the responsibility of others, mostly the partner or the gastronomy. While individuals of The Unintentionally Restraining Type do not actively engage with and tend to question the benefits of RDPs, some participants belonging to this type showed a certain degree of readiness to refrain from harmful foods—if those were declared as such by an authority:

*“Yes, if there was a warning published by the public health service or by the government, I would stop doing it, yes. Yes, indeed.”* (7M).

Yet, such a readiness was only expressed upon explicitly being asked by the interviewer—unlike the other types, where such objectives were expressed on the participants’ own initiatives. In terms of the health context, Type IV may potentially convert to Type III for the purpose of primary or secondary prevention. Above such a potential avoidance of officially harmful products, no further motives surfaced. Congruent with the limited, unintentional RDP, no barriers to the implementation of restrictive dietary practices were evident for this type.

## 4. Discussion

This qualitative study explored Restrictive Dietary Practices (RDPs) and related attitudes towards as well as motives for and barriers to its implementation in a sample of middle-aged and older German adults. Using qualitative content analysis [35], several themes and (sub-)categories inductively revealed, based on which four types of RDPs could be reconstructed.

Individuals assigned to the Holistically Restraining Type (Type I) were characterized by their holistic integration of RDP as part of an implemented concept of a healthy diet based on profound food knowledge and habitual engagement in foodwork. Intrinsic motives were based on a general health orientation, but also on ethical and ecological attitudes. In contrast to Type I, dietary restraint thereby did pose an emotional constraint for individuals belonging to the Dissonant-savoring Restraining Type (Type II), which indeed accepted the necessity to refrain from certain foods in order to stick to a healthy diet. However, they were not willing to renounce from certain foods since this would entail a limitation in the enjoyment food and the freedom of choice integral for their quality of life. The Reactively Restraining Type’s RDP (Type III) was characterized by a clear health-related goal in the face of manifest illness or risk factors. Moreover, this type only refrained from particular selective foods. Compared to the first three types, for the Unintentionally Restraining Type (Type IV), dietary restriction was not a conscious practice and solely referred to as an externally triggered necessity if foods were officially declared to be harmful.

Although differences between dieting and restrictive eating have been proposed [8], our typology is situated well against the background of Barlösius’s broader categorization between two dieting styles that involve RDPs [36]. The first type of dieting is seen as a disease therapy of or measure for weight reduction based on recommendations by medical experts, such as physicians and dieticians, which is relatable to individuals belonging to Type III. In contrast, Barlösius also refers to a stringent dietary restriction, which is not considered as a diet or (only) based on scientific facts. Instead, it is integral to a holistic lifestyle concept based on, for instance, particular world views or philosophies, such as anthroposophy. In our study, this was characteristic for some individuals belonging to Type I. Exceeding these previous findings focusing on the initiation of RDP, the typology of our study offers a broader view on RDPs involving the actual implementation of RDPs and the associated barriers to do so as well as the attitudes and motives underlying such practices.

### 4.1. Type Shiftings in Changing Lifeworlds

Regardless of the description of differing characteristics in this typology, a partial overlap as well as dynamic shift between the types is possible. Such shifts are likely, since dietary practices are not only subject to various (primary, secondary, and tertiary) socialization processes [37] and thus constantly affected by a complex of individual–biographical and socio-environmental influences [38]. Therefore, practices such as those related to food may need situational (re-)adjustments in accordance with the individual’s changing lifeworld, needs, and resources. Particularly a conversion into Type III from any of the other types but also other ways of type-shifting seem likely in the context of a biographical transition or upheaval. Previous research has shown various changes in diet-related practices, including RDPs, in relation to the onset of a(n) (severe) illness [39], changes in partnership status (e.g., widowhood) [40,41], as well as to the transition to retirement [42], for instance. As another example, a shift from Type I towards Type IV seems rather unlikely than the inverse case, as knowledge that once has been acquired does remain and deeply grounded convictions and intrinsic motives are less prone to be neglected.

### 4.2. Modi Operandi of and Motives for RDP

Our typology shows that—to differing extents—all study participants reported on practices of dietary restraint. In our sample, the modi operandi of RDPs were mainly distinguishable with respect to (a) the timeframe and (b) the targets or ‘objects’ of restriction. With respect to the former (a), the degree to which dietary restraint was practiced differed from “rigid” to “flexible”, which is congruent with the definitional differentiation that has previously been (quantitatively) derived [16]. In that context, the regularity, intervals as well as the amount of time spent on RDPs appeared to be crucial. The regularity of restriction ranged from never or occasional to continuous and flexible versus a strict habitual implementation into daily routines. Interval fasting was one main modus operandi and the execution of specific “diet days” another. Moreover, some participants described having practiced dietary restriction for years, and others had just currently changed their diets or were planning on doing so.

Regarding (b) the targets of RDPs, participants restrained, on the one hand, from specific foodstuffs or nutrients, such as those rich in refined carbohydrates or fats. On the other hand, the targets of RDPs were single foods or drinks, for instance, sugar, alcohol, or meat. The restriction of meat consumption was a major theme that was raised without exception across all interviews. We found a widely ranging degree of restriction, in which specifically women referred to various practices of restricted meat consumption. There is a large body of literature on the meaning of meat in the re-constructing norms of masculinity and traditional gender roles [43,44,45,46], against the background of which our findings may be interpreted. The intentions to refrain from meat mentioned in the research to date [22,47,48] also comply with our findings on motives underlying RDPs that are related to health promotion and ethical as well as environmental convictions. Interestingly, while our participants did mention animal welfare and the preservation of the environment and natural resources as reasons to refrain from meat (among others) consumption, no one referred to its potential detrimental effects on climate change. This is congruent with other research findings that underline an overall lacking awareness on the effects of food choices on the climate as well as the symbolic and cultural importance of meat, among other things [49,50,51].

Despite those related to meat consumption and vegetarian diets, the predominant motives to engage in RDPs in our sample were health promotion and regulation as well as weight-management or control, which are typical for “restrained eaters” [33] as previous research has found.

### 4.3. Barriers and Reluctance to adopt RDPs

In the literature, dietary restraint as a cognitive construct has been differentiated as “successful” and “unsuccessful”, referring to its actual practical implementation [16,17]. In our study, a reluctance to restrain from foods primarily planned to reduce or exclude from the individual’s diet and therefore “unsuccessful dietary restraint” was shown in the practice of several participants of Type II. In some cases, the reluctance found expression in an explicit, conscious decision not to restrain (partly Type II and Type IV). Such ‘barriers’ were explicitly mentioned to be spontaneity and the rejection of rules in order to maintain the quality of life. The savoring of food was referred to by all types to interfere with restrictive practices. However, especially in the case of Type I, exceptions of RDPs were not regarded as failure but within a set frame of, for example, social integration. Indirectly, this has been described, for example, by another qualitative study reflecting on the motivations of different dietary patterns, where the predominant motivation of an omnivore diet compared to vegan or vegetarian diets was declared to be taste and enjoyment [22].

In the context of caloric restriction, previous work indicated increased appetitive hunger signals due to hormonal regulation to be a barrier to refrain from eating [52]. This manner of RDP was implied amongst individuals of Type I, who expressed the occurrence of hunger, but no behavioral consequence, and Type II participants who shared their experiences of relapse. The same study [52] revealed ‘rule rigidity’ as a central barrier to caloric restriction, which was also explicitly mentioned as such in our study by Type II and Type III. Furthermore, time-restricted eating was described to be abandoned due to the availability of foods and the effort of scheduling and limiting the timely window of eating. Performed only by individuals of Type I, these aspects did not appear as barriers from our data.

### 4.4. Restrictive Dietary Practice as a Social Norm and Means of Distinction

From a praxeological perspective, (sub-)conscious orientations and attitudes manifest themselves in practices, such as RDP, and—to speak with Bourdieu [53]—therefore build a person’s habitus, which is closely related to an individual’s milieu. Thereby, practices serve the function of social distinction to (re-)produce social difference and, hence, to secure one’s (superior) social position. With respect to dietary styles, a specific diet and, therefore, some kind of a restrictive dietary practice is based on knowledge and is often morally justified. Particularly, individuals with a middle class status and a respective educational background reveal significantly higher engagement in reflected and conscious (e.g., regarding sustainability) dietary practices [53].

Meat with its highly symbolized and changing social meaning is a prominent example of this [36,54] and also was as a central object of restraint in our study. While meat used to be a privilege of the upper class, it became a mass-consumed product since WW2, but (high) meat consumption—especially in view of discourses on climate change and health orientation—has increasingly become negatively connotated [55]. Thus, in certain milieus, such as the middle class, as in our sample, refraining from meat signifies social distinction. This may be because, for example, a restricted meat consumption may symbolize a deeper reflection on the social and environmental consequences related to the individual’s behavior and underscores his/her responsible action.

Moreover, in our sample, social distinction also revealed with respect to the individual’s self-responsibility for a healthy life(style). As has previously been argued, the adherence to any form of dieting may demonstrate some kind of social distinction as long as the decision is not the consequence of a medical necessity [36]—which we saw in Type III of our typology. More specifically, Type II individuals expressed their health orientations, since they—contrary to Type IV—showed elevated levels of health- and diet-related knowledge and were explicitly willing to make an effort for health promotion and weight control. The ability to restrain from leisure in the context of food symbolizes normative values of self-control and moderation. These are personal qualities that are often associated with a generally systematic, regulated lifestyle [36] and are imperative to the 21st-century developments and norms of individualization and self-responsibility [56].

### 4.5. Restrictive Dietary Practices as a Starting Point for Health Promotion

As mentioned previously, most guidelines on a healthy diet involve RDPs. The recommendations published by the World Health Organization [6], for example, suggest a reduced or moderate intake of sugar, processed fats, and salt. Aside from the knowledge required to follow such recommendations, adhering to them poses a major challenge, as illustrated by the much-cited “intention–behavior gap” [57,58].

In our study, a divergence between intention and behavior could be seen in the cases of “unsuccessful dietary restraint” occurring predominantly in the practice of some participants assigned to Type II. Moreover, certain food environments, such as those in most Western industrialized countries—where ubiquitous food offerings and advertising encourage overconsumption—constitute hampering conditions for the adherence to a healthy, and thus partly restrictive, diet [59]. At the same time, dieting and restraint have increased in recent decades—which is certainly shaped by socially normalized body images and ideals of thinness and can be framed as a countermovement to the so-called obesity epidemic [60,61,62,63].

Irrespective of the background and depth of diet-related knowledge, the restrictive practice is aligned with general recommendations as those, for example, published by the WHO [6], which include, in addition to the daily consumption of vegetables and fruits, the replacement of refined carbohydrates by wholegrain foods and limit the consumption of sugar, processed foods, and fatty meat. The only exception is the recommendation of reduced salt consumption, which did not emerge in the interviews. These recommendations imply RDPs to be a central intervention point for health promotion. The amelioration of objective health parameters was expressed predominantly by individuals of Type III and Type I. Primary and secondary prevention were also among the motives of Type I and Type II. Individuals of Type III in particular, revealed the amelioration of objective health parameters. This could already lay a foundation for public health measurements that may build up on established restrictive practices or guide towards a healthy diet. For individuals assigned to Type II and Type III, these might include to promote risk communication and evaluation between long-term risks and short-term pleasure of consuming unhealthy foods. Likewise, for Type IV, this seems to be a necessary strategy preceded by the education of the effects of diet on health in general. Restrictive dietary practices could also be promoted by limiting the accessibility of harmful foods, which would, for example, reduce temptations for Type II or render the unconscious consumption of unhealthy foods for Type IV less likely.

Especially in the age group of the study participants, the public discourse should be promoted, as RDP depicts to be effective even at older age. Furthermore, dietary restraint has been reflected on as a healthy self-regulation mechanism, including self-monitoring, realistic goals, and self-evaluation that promotes successful weight management [7,8].

### 4.6. Strengths, Limitations, and Future Research Directions

This is the first study to qualitatively reconstruct different types of dietary restraint practices among a general, non-clinical sample of middle-aged and older adults. Our findings provide insights into the complex mechanism between practice, associated attitudes, and motives for as well as the reasons for a reluctance towards and so-called “unsuccessful” RDP. Therefore, we fill an important research gap and add a nuanced understanding of a central aspect of everyday dietary practices to the existing body of knowledge. Moreover, one strength of our study is the methodological approach. To date, the debate on the assessment of RDPs has focused on quantitative measures, neglecting the importance of reconstructive qualitative methods to gain a deeper understanding of the subject of interest. Previous classifications have focused on objects that were refrained from, while no previous work could be found that investigated the general implementation and degree of a RDP with a focus on this age group. In the present study, a spectrum of different manifestations could be identified, clustering the consumers’ behaviors with a broader view towards various aspects subordinating the objects of restraint. The theme of RDP emerged immanently from the data material, i.e., participants were not asked about restrictive dietary behavior or dieting. Thus, the participants’ partly implicit, partly explicit emphasis on dietary restraint underscores its relevance to their lifeworlds. This may certainly have reduced the bias of social desirability.

At the same time, the recruitment procedure may have deterred potential participants due to feelings of not matching a social desirability or a lack of interest in the study’s topic. Although we could observe a wide range of engagement in adopting RDPs, it should be considered that individuals with a greater interest in and involvement with diet-related topics might have been more likely to take part in our diet-related study. Moreover, the fact that the majority of our participants was female can be regarded as a potential bias towards a higher engagement in Restrictive Dietary Practices, which is consistent with previous work [64]. Furthermore, as described above, although assigned to one of the four types of our typology, not only a dynamic shift between the types is possible, but furthermore timeframes may partially remain vague. Defining Type I’s motivation as intrinsic and the integration of restrictive practice as a long-term habit, for example, might be the result of a lack of information acquired in the interview, as it might just as well had been external triggers that led to the present practice. Therefore, it should be taken into consideration that all results are based on a snapshot of the individual’s situation and momentary recollection during the interview. This indicates a necessity for more longitudinal data.

Moreover, future studies that investigate RDPs and our typology with respect to health-related parameters (e.g., disordered eating styles (e.g., binge eating), disease burden, subjective wellbeing, and health-related quality of life) to measure potential (adverse) health effects of RDP are worthwhile. Only in some cases of Type III and Type I, the amelioration of objective health parameters was named as the result of a successful RDP. More generally, further investigations of RDP (among older adults) are promising with respect to individual–psychological factors, such as diet-related self-identifications and the respective social positioning (e.g., as “picky eater”) [65], on the one hand. Analyzing various personality traits and characteristics, such as self-discipline or cognitive flexibility, regarding changing and (re-)establishing dietary habits of RDPs thereby appears worthwhile. Systematically examining the social circumstances and determinants that may influence such behavioral or psychological patterns associated with RDPs will be the task of future (quantitative) studies. In addition, multiple political as well as social orientations and values appear relevant in shaping RDPs. For instance, important insights may be gained by researching dietary restraint (among older adults) in the context of feminist identity and/or the norms spread by (late-modern) societal developments [66,67,68].

Finally, the transferability of our findings needs to be discussed. Crucially, the socio-cultural context within which our study was carried out needs consideration. Since cultural norms and specific food environments, amongst others, shape dietary practices in general, the mechanisms of restrained eating also vary across ethnic groups [36,69]. A similar case is that of a complex of social determinants, including class belonging, influence dietary behavior, and thus RDPs [70,71,72]. Our sample consisted of German adults that were generally highly educated and have middle incomes, and thus did not appear to be concerned with food insecurity, which has previously been shown to affect RDPs [73]. Hence, future studies are needed that investigate RDPs, for example, in non-industrialized countries as well as more vulnerable population groups. Moreover, it is the task of future quantitative and particularly mixed-method investigations to statistically validate our findings against the background of various social determinants.

With respect to societal developments, our interviews were conducted before the COVID-19 pandemic and the worldwide significant rise in inflation rates. While the latter may certainly have an impact on household spending—particularly in lower income classes—the COVID-19 pandemic has shown to have (moderate) effects on dietary changes among older adults [74]. Hence, both aspects may have affected the ways in which our target group was engaged with RDPs. At the same time, there has been a steady increase in the public debate on climate change and the effects of diet-related behavior (e.g., fostered by movements, such as Fridays for Future or Extinction Rebellion). Closely related, (German) production rates and consumption patterns of vegetarian as well as vegan animal-based alternatives, for instance, have increased and hence indicate changes in RDPs [49,75]. Against the background of these developments, motives for and the extent to which RDP is implemented into daily routines might have changed within recent years (e.g., in the context of the COVID-19 pandemic). While our typology is conceivable to be still applicable, it is the task of future investigations to further explore the relation between such societal changes and RDPs in middle-aged and older adults.

## 5. Conclusions

We showed that Restrictive Dietary Practice (RDP) was a substantial and common topic mentioned in the interviews regarding dietary practices among middle-aged and older adults. The qualitative and quantitative extent to which dietary restriction was practiced differed on a wide scale. The central motives that were revealed by this study where health, well-being, weight control, and ecological and ethical reasons. The major barriers that were referred to were the enjoyment of foods and the willingness to maintain a spontaneity that allows the choice of food disregarding any rules of restraint. The understanding of RDPs has clinical relevance, especially in the context of health promotion. In a surrounding of an omnipresent food supply and temptations leading to a consumption of foods and amounts of energy deleterious to health, overeating is a central health, social, and economic issue of wealthy societies. Deliberate restriction, therefore, becomes an individual and societal necessity and depicts to be efficient even in populations of advanced age. In order to foster this behavior, it is necessary to understand the present implemented practices, motives, and barriers, which we investigated in our study. As our findings indicate, the levels of food-related knowledge were not only diverse, but essentially, the base for conscious choices of restriction. Apart from specific health benefits RDPs have to offer, the capacity to self-regulate and self-empowerment itself may promote individual wellbeing and may therefore contribute to encouraging behavioral changes in general. Therefore, successful and health-related RDPs are important to be considered as a foundation for the promotion of health-orientated self-efficacy and point of intervention for disease prevention and management.

## Figures and Tables

**Table 1:** Major interview themes and sample guiding questions (exmanent and immanent).

Narrative stimulus—socialization background	“Please tell me about how you grew up. When you think about your youth/childhood, what comes into mind?”
Dietary routine in childhood	“Was there a daily routine of eating together with the family?” “What did you eat?”
Current lifestyle	“Please tell me about yesterday. What did you do?” What do you do at weekends? Can You describe what you did last weekend?”
Diet in daily life and at special occasions	“When you think about last Christmas, how did you celebrate? What did you eat? Who prepared the food? What is different to your daily dietary routine?”
Readiness for dietary change	“Have you ever changed your diet? And if so, what was your motive and did you succeed? Do you currently have similar ambitions for the future? Please tell us about that.”

Note. Guiding questions were posed in the German language in the interviews.

**Table 2:** Participant characteristics.

#	GENDER	AGE (YRS.)	INTERVIEW LENGTH (MIN.)	RELATIONSHIP STATUS	PROFESSION	VOCATIONAL STATUS	RESTRICTING TYPE
**W1**	female	63	70	single (divorced)	pedagogue/pediatric nurse	secondary school certificate	3
**W2**	female	62	75	single (divorced)	proofreader	general qualification for university entrance	1
**W3**	female	60	50	single (widowed)	laboratory assistant	secondary school certificate	1
**W4**	female	64	77	single (divorced)	nurse	technical school certificate	3
**W5**	female	62	46	single (widowed)	medical technical assistant	technical school certificate	1
**M6**	male	78	81	married	police officer	advanced technical college certificate	4
**M7**	male	60	51	partnered (separately living)	taxi driver	general qualification for university entrance	4
**W8**	female	66	72	married	cosmetician	secondary school certificate	2
**W9**	female	61	59	married	computer scientist	advanced technical college certificate	2
**M10**	male	68	65	married	administration secretary	secondary school certificate	2
**M11**	male	69	73	single (divorced)	interpreter	university degree	3
**W12**	female	69	66	single (widowed)	photographer	secondary school certificate	3
**W13**	female	67	72	married (separately living)	stenotypist	secondary school certificate	2
**W14**	female	68	76	single	stenotypist	secondary school certificate	3
**M15**	male	59	62	single (divorced)	taxi driver, mechanic	polytechnic secondary school	3
**W16**	female	68	73	partnered	engineering draftsman	technical diploma	1
**M17**	male	60	62	married	clerk	secondary school certificate	3
**W18**	female	66	88	single (divorced)	librarian	university degree	3
**M19**	male	69	87	single (divorced)	(vocational) teacher	university degree	1
**M20**	male	65	71	partnered	journalist	general qualification for university entrance	2
**W21**	female	64	77	partnered (divorced)	wholesaler	secondary school certificate	2
**W22**	female	65	88	married	teacher	university degree	2
**W23**	female	76	67	single (divorced)	seller, prev. self-employed	secondary school certificate	2
**M24**	male	61	52	single (divorced)	executive employee	university degree	3

Note. # = interview pseudonym; yrs. = years; min. = minutes; prosp. = prospectively; restricting type = inductively generated typology classification: Type 1 =The Holistically Restraining Type, Type 2= The Dissonant-savoring Restraining Type, Type 3 = The Reactively Restraining Type, Type 4 = The Unintentionally Restraining Type.

**Table 3:** Characteristics of the typology of restrictive dietary practice (RDP).

	I. The Holistically Restraining Type	II. The Dissonant-Savoring Restraining Type	III. The Reactively Restraining Type	IV. The Unintentionally Restraining Type
**(1) Practical implementation of RDP**	- Long-term, internalized habitual practice of RDP; - “Ascetic” in terms of certain foods (vegetarian) or amount of energy (low-calorie-days); - within a certain timeframe (intermittent fasting).	- General tendency towards RDP; - Flexible practice; - Exceptions of RDP for the enjoyment of food.	- Restriction of specific foods or food components.	- Minor/no conscious restrictive practice; - Food choice that excludes foods due to, e.g., preference or habits.
**(2) Attitude towards RDP** - **Benefits regarding health and wellbeing** - **Individual Integration of RDP**	- Positive, based on personal experience and attribution of benefits concerning wellbeing and health; - RDP evokes no emotional constraint; - Conviction and integration of a holistic concept of life (includes RDP regarding foods considered unhealthy).	- Positive, based on personal experience and attribution of benefits concerning wellbeing and health; - Perception of health benefits (primary prevention: avoidance of physical illness); - Distancing from rigid RDPs as a threat to quality of life; - Desire to maintain freedom of (food) choice; - Enjoyment competes with reason; - Orientation towards a healthy diet (balanced, not a specific philosophy or trend).	- Positive; - Perception of specific health benefits (e.g., regarding secondary prevention: reversal of or overcoming disease); - Distinction to practice based on (internalized) rule.	- Questioning of benefits; - Vague readiness to adopt a RDP if recommended by authorities (e.g., government/physician).
**(3) Motives for RDP**	- Health/wellbeing: central motives - Weight control: central motive - Environmental aspects: central motives: - - Avoidance of pesticides, preservation of resources; - Rejection of meat production; consideration of animal welfare;	- Health/wellbeing: Central motives (primary prevention; avoidance of physical illness); - Weight Control: central motive; - Environmental aspects: secondary motive.	- Health: (secondary prevention, reversal, or stop of physical illness, extrinsic motive (e.g., recommended by physician); - Weight control: central motive; - Environmental aspects: not addressed.	- Health (avoidance of strongly hazardous when officially recommended); - Weight control and environmental aspects: not addressed;
**(4) Barriers towards RDP**	- Enjoyment of food. - However, reflection and control of circumstances (e.g., availability of foods); - Exceptions of restrictive practice not considered as failure, but deliberate (e.g,. Social events).	- Enjoyment of food; - Psychological health and quality of life are threatened by rigid dietary restraint; - Essential need for freedom/spontaneity.	- Enjoyment of food; - Pressure to follow rigid rules of RDP perceived as counter-productive.	- No perception of a barrier (no primary intention to restrain); enjoyment excludes the possibility of restraining from foods; - Dietary routine that excludes foods not included in habitual food choice.

## Data Availability

Anonymized primary data (in the German language) are available upon request from the last author.

## Appendix A

**Table A1:** Category system with the emergent major themes, (sub)themes, and exemplary manifestations.

Major Theme	
Restrictive Dietary Practice (RDP)	
**(Sub)themes**	Exemplary Manifestations
**Attitudes towards RDP**	* positive * negative * RDP as societal guiding principle * theoretical readiness for RDP * RDP as plausible reaction to food scandals * rejection
**Practical Implementation of RDP**	* biographical context of RDP * object of restriction * degree of restriction * no conscious RDP * explicitly no RDP
**Motives for RDP**	* health * weight control * weight loss * well-being * animal welfare * preservation of natural resources/environment * gain of self-control * social integration* taste aversion
**Barriers towards RDP**	* savoring of food * need for spontaneity * rejection of rules * social integration * availability of foods
